# Galectin-Receptor Interactions Regulates Cardiac Pathology Caused by *Trichinella spiralis* Infection

**DOI:** 10.3389/fimmu.2021.639260

**Published:** 2021-05-21

**Authors:** Jinghai Yan, Shiguang Huang, Fangli Lu

**Affiliations:** ^1^ Department of Parasitology, Zhongshan School of Medicine, Sun Yat-sen University, Guangzhou, China; ^2^ Key Laboratory of Tropical Disease Control of Ministry of Education, Sun Yat-sen University, Guangzhou, China; ^3^ School of Stomatology, Jinan University, Guangzhou, China

**Keywords:** *T. spiralis*, mice, myocarditis, galectin-receptor interaction, Gal-3, eosinophils

## Abstract

The parasitic nematode *Trichinella spiralis* causes trichinellosis, a serious food-borne parasitic zoonosis worldwide. Infection with *T. spiralis* may also cause myocarditis. In the present study, we used mouse models to assess the impact of blockage of galectin-receptor interactions by α-lactose on cardiac immunopathology during acute *T. spiralis* experimental infection. Our data demonstrated that, after *T. spiralis* infection, blockage of galectin-receptor interactions resulted in cardiac dysfunction detected by transthoracic conventional echocardiography, and increased serum Gal-3 level, a biomarker of myocardial damage. In addition, there were increased eosinophil number in peripheral blood, and increased eosinophil infiltration in the heart and spleen tissues accompanied with increased mRNA levels of eosinophil granule proteins (including eosinophil cationic protein (ECP) and eosinophil peroxidase (EPO)) and IL-5 in these organs; increased cardiac fibrosis accompanied with increased Gal-3 and collagen 1 expressions in the hearts of mice with blockage of galectin-receptor interactions after *T. spiralis* infection. Correlation analysis showed that significant positive correlations existed between the mRNA levels of Gal-3 and ECP/EPO/eosinophil major basic protein/IL-5/CCL11/CCR3/α-SMA/collagen 1 in the hearts of both *T. spiralis*-infected mice and *T. spiralis*-infected mice with blockage of galectin-receptor interactions. Our data suggest that galectin-receptor interactions play a pivotal role during acute *T. spiralis* infection, and lack of galectin-receptor interactions upregulates Gal-3 which, in turn, leads to elevated heart eosinophil recruitment, exacerbated heart pathology and fibrosis, and heart functional damage.

## Introduction

Trichinellosis is one of the most important foodborne parasitic zoonoses caused by nematodes of the genus *Trichinella*, which are worldwide distributed, including Europe, Southeast Asia, North and South America, and North Africa (1). Cases of trichinellosis have been reported worldwide except for Antarctica (2–4). *Trichinella spiralis* species is the common cause of human disease by eating raw or undercooked pork (5, 6). Though *T. spiralis* causes muscle pain almost without life risk, myocarditis and neurological disorders is the most serious complication of human infestation by *T. spiralis*, and myocarditis that may lead to death without treatment (7). When *T. spiralis* parasitized in the host, it can induce host’s immune response and high numbers of eosinophils in the peripheral blood, which play an important role in defense against infestations. Eosinophils also increased in the epithelium of the duodenum of animal models at 10 days after *T. spiralis* infection (8). Eosinophilic myocarditis is a severe complication of trichinellosis that can lead to death due to rhythm disorders (9). However, so far the pathological mechanisms of eosinophilic myocarditis caused by *T. spiralis* remains poorly understood.

The eotaxin family includes three members: CCL11 (eotaxin-1), CCL24 (eotaxin-2), and CCL26 (eotaxin-3) (10). It has been reported that IL-5 is increased in mice infected with *T. spiralis* (11). CCL11 and IL-5 act synergistically to stimulate the release of eosinophils from bone marrow and recruit eosinophils into inflammatory sites (12). Eosinophils are recruited to the inflamed area in response to stimuli, regulating the immune response through the release of their granule proteins (13). Eosinophil peroxidase (EPO), which can generate potent oxidizing species, is the most abundant cationic protein of the matrix of secondary granules. It not only produces proinflammatory oxidants but also plays a cytotoxic role as a cationic toxin against both parasites and mammalian cells (14). Eosinophil cationic protein (ECP), one of the cationic granule proteins, is a ribonuclease, which has marked toxicity for a wide variety of helminths, bacteria, single-stranded RNA viruses, and host tissues (15, 16). ECP can be secreted into the extracellular area in an antibody-dependent and an antibody-independent manner (17, 18). Eosinophil major basic protein (MBP) is among the most abundant proteins in eosinophils, and the toxicity of MBP plays an important role in eosinophil protection against parasitic infections (19, 20).

Galectins are proteins that bind *β*-galactosides, and have diverse roles in inflammation, immune response, cell migration, and signaling pathways (21, 22). Host galectins have been shown to modulate the effector function of mast cells, neutrophils, and eosinophils (23). Most galectins distribute intracellularly and extracellularly and extracellular galectins combine with glycans on the cell surface and induce various cellular responses (24). The major galectins expressed in the heart are galectin (Gal)-1 (25) and Gal-3 (21). Gal-1, a potent anti-inflammatory and immunoregulatory molecule, plays a role in various immune or inflammatory diseases, like acute myocardial infarction (25). Gal-1 also prevents infection and damage caused by *Trypanosoma cruzi* on cardiac cells (26). Gal-3 has a unique structure, e.g. an extended N-terminal peptide and a C-terminal carbohydrate-recognition domains (21). As a biomarker in heart failure and cardiovascular diseases, Gal-3 is an important modulator of biological processes and an emerging player in heart inflammation and fibrosis (27). Gal-9 is mainly expressed by eosinophils, T cells, endothelial cells, kupffer cells, DCs, macrophages, vascular endothelial cells, and intestinal epithelial cells (28). Gal-9 in combination with rapamycin can induce cardiac allograft tolerance (29). In addition, *T. spiralis* galectin (Tsgal) has been identified in the muscle larva surface protein (30, 31), rTsgal protein may promote the larval invasion of host’s intestinal epithelial cell monolayer, while the anti-rTsgal serum inhibits the larval invasion of the monolayer in dose-dependent mode (32). It has been reported that manipulating galectin signals in mice can be achieved using α-lactose (33).

Among the complicated fibrosis signaling networks, the TGF-β1/α-SMA/collagen 1 profibrotic pathway has been widely recognized to induce cardiac hypertrophy and fibrosis in the failing heart (34). In this study, we used mouse models to investigate whether galectins involved in the immunopathological mechanisms of myocarditis caused by *T. spiralis* infection through blocking galectin-receptor interactions with α-lactose, our data found that Gal-3 and eosinophils play important roles in the cardiac pathology and fibrosis caused by *T. spiralis* infection.

## Material and Methods

### Ethics Statement

Animal experiments were approved by the Animal Experimentation Ethics Committee of Zhongshan School of Medicine on Laboratory Animal Care at Sun Yat-sen University (No. SYSU-IACUC-2019-B077), and carried out in strict accordance with institutional Guidelines for Care and Use of Laboratory Animals.

### Mice, Parasites, and Infections

Female, 6-8 weeks-old Kunming mice were purchased from the animal facility at Sun Yat-sen University in Guangzhou, China. *T. spiralis* (pig strain) was maintained in our laboratory *via* serial passage in mice, and the larvae were recovered from muscles of mice at 60–90 days post-infection (p.i.). Standard procedures were used for isolation, collection, and inoculum (35). A total of 40 mice were used in this experiment. Mice were randomized into 4 group*s* with 10 in each group, (i) uninfected control group; (ii) α-lactose-treated group: mice were injected with α-lactose alone; (iii) *T. spiralis-*infected group: mice were infected with 300 *T. spiralis* larvae by oral gavage, and (iv) *T. spiralis +* α-lactose group: mice were infected with 300 *T. spiralis* larvae and with α-lactose treatment. Some mice were injected intraperitoneally with 1.5 mM of α-lactose solution in phosphate buffer saline (PBS) once daily starting from 1 to 15 days p.i. Mortality of each mouse was monitored daily. All the mice were sacrificed at day 15 p.i. and their organs were taken for further analysis at 12 hours after the last treatment.

### Peripheral Blood Eosinophil Count

On day 15 after *T. spiralis* infection, thin blood smears of peripheral blood of different groups of mice were stained by Wright-Giemsa staining, and eosinophils were counted per 100 karyocytes under 1000× oil-immersion light microscopy.

### Measurement of Serum Cardiac Troponin T (cTnT) and Gal-3

Mice of different groups were euthanized by CO_2_ asphyxiation on day 15 after *T. spiralis* infection, blood of each mouse was collected and allowed to clot for 6 h at 37°C, and centrifuged at 3000 × g for 10 min to separate serum. After centrifugation, the serum was collected and stored at −80°C until further use. Different ELISA kits were used to measure serum levels of cTnT (Solarbio Life Science, Beijing, China) and Gal-3 (Boster Biological Technology, Wuhan, China) to determine the cardiac damage. OD values at 450 nm were recorded using the microplate reader (Sunrise, TECAN Austria).

#### Echocardiography

On day 30 after *T. spiralis* infection, transthoracic conventional echocardiography was performed using a Vevo 3100 Imaging System (Fujifilm VisualSonics, Toronto, Canada) employing a MS550D transducer with a center frequency of 40 Hz and an axial resolution of 40 µM. For this examination, hairs on the chests of mice were removed. Twenty-four hours later, mice were anesthetized with 1-3% isoflurane (Ezvet, Beijing, China) and taped in the supine position on a heated 37°C pad. Anesthesia was maintained with 0.5-3% isoflurane *via* a nose cone. M-mode images were acquired from parasternal short axes views to assess left ventricular (LV) percent ejection fraction (LVEF), fractional shortening (FS), stroke volume (SV), and cardiac output (CO).

### Histopathological Analyses

Mice were euthanized by CO_2_ asphyxiation on day 15 after *T. spiralis* infection, and heart and spleen were harvested and immediately fixed in 10% buffered natural formaldehyde (Guangzhou Chemical Reagent Factory, Guangzhou, China) for over 48 h. Four-micrometer-thick serial tissue sections of the organs from each mouse were stained with hematoxylin and eosin (H&E) (Sigma-Aldrich, St. Louis, MO, United States) and imaged under light microscopy. To evaluate the histological alteration of heart and spleen, a semi-quantitative scoring system was used. The histopathological changes of heart tissue from each mouse were determined under 400× or 1000× magnification in three noncontiguous sections. Microscopic scores of the severity of inflammation were graded into four grades: 0, no inflammation; 1, presence of a few small lesions, not exceeding 0.25 mm^2^ in size; 2, presence of multiple small lesions or a few moderately sized lesions, not exceeding 6.25 mm^2^; and 3, presence of multiple moderately sized lesions or more larger lesions (36). The numbers of eosinophils in heart and spleen tissues were quantified using images captured with a digital camera system under 1000 × magnification and analyzed by using Image-Pro Plus (version 6.0, Media Cybernetics, Inc., MD, United States), and the density of eosinophils was expressed as the number of eosinophils per square millimeter.

### Sirius Red Staining

To detect the deposition of collagen fiber in heart tissues, paraffin-embedded heart from each mouse was sectioned at 4 µm and stained by sirius red stain kit (Beijing Leagene Biotchnology Co., Ltd., Beijing, China). The positive areas of fibrosis of heart tissues were quantified using images captured with a digital camera system under 400 × magnification and analyzed by using Image-Pro Plus 6.0 (Media Cybernetics, Inc.).

### Immunohistochemical Staining

The frozen heart of each mouse was covered with optimal cutting temperature compound, and 10-µm-thick serial heart tissue sections were washed with PBS. Heat-induced antigen retrieval was carried out in an 800-W microwave oven for 30 min. Sections were treated with 3% hydrogen peroxide in methanol for 10 min at 37°C, and then incubated in 5% bovine serum albumin (BSA) in PBS (pH = 7.4) for 10 min at room temperature to block nonspecific binding. After washing with PBS, sections were incubated with rabbit anti-Gal-3 (1:200 dilutions) (Boster Biological Technology) overnight at 4°C. Those sections incubated with secondary antibodies alone were used as isotype controls. Immunohistochemical staining was then performed with a streptavidin–biotin–peroxidase complex kit and developed with diaminobenzidine tetrahydrochloride (Zhongshan Golden Bridge Technology, Beijing, China). The sections were counterstained with hematoxylin and positive cells were identified by dark-brown staining under light microscopy. The signal with immunohistochemistry (positive areas) of Gal-3 were quantified using images captured with a digital camera system under 400 × magnification and analyzed by using Image-Pro Plus 6.0 (Media Cybernetics, Inc.).

### Immunofluorescence Staining

To detect extracellular trap formation, frozen sections of the hearts from mice of different groups were fixed with 4% paraformaldehyde for 15 min at room temperature. After washing with PBS, the slides (10-µm) were blocked with 5% BSA for 1 h, and then were incubated with Hoechst 33342 (Sigma-Aldrich) for 5 min at room temperature to stain DNA. All the slides were analyzed by a fluorescence microscope (BX63, Olympus, Japan).

### Determination of mRNA Expression by Using Quantitative Real-Time Reverse Transcription-Polymerase Chain Reaction (qRT-PCR)

Total RNA was extracted from about 100 mg of heart and spleen tissues of each mouse using a RNA Extraction Kit (TaKaRa Bio, Inc., Shiga, Japan) as per the manufacturer’s protocol. RNA amount was determined by measuring the ratio of absorbance at 260 and 280 nm using a NanoDrop ND-1000 spectrophotometer (NanoDrop Technologies, Inc., Wilmington, DE, United States). First-strand cDNA was constructed from 1.0 µg of total RNA with oligo (dT) as primers using a PrimeScript 1st Strand cDNA Synthesis Kit (TaKaRa Bio, Inc.). To determine tissue mRNA levels of galectins (Gal-1, Gal-3, and Gal-9), eosinophil cationic granule proteins (EPO, ECP, and MBP), chemokines (IL-5, CCL11, CCL24, and CCR3), and β-actin in both heart and spleen, and TGF-β1, α-SMA, and collagen 1 in heart, qRT-PCR assay was performed using SYBR Green QPCR Master Mix (TaKaRa Bio, Inc.). Primers are listed in **Table 1**. Briefly, a total of 10 µl reaction mixture contained 5.0 µl of SYBR^®^ Premix Ex TaqTM (2×), 0.5 µl of each primer (10 pM), 2.0 µl of cDNA (0.2 µg/µl), and 2.0 µl of ddH_2_O. Amplification was pre-denaturized for 30 s at 95°C, followed by 39 cycles of 5 s at 95°C and 30 s at 60°C using a CFX96 Touch^®^ Real-Time PCR Detection System (Bio-Rad Laboratories, Hercules, CA, United States). The mRNA expression levels of galectins (Gal-1, Gal-3, and Gal-9), eosinophil cationic granule proteins (ECP, EPO, and MBP), chemokines (IL-5, CCL11, CCL24, and CCR3), TGF-β1, α-SMA, and collagen 1 were normalized to that of mouse housekeeping gene, β-actin. The results were expressed as fold change compared with uninfected controls.

**Table 1:** Primer sequences of genes used for quantitative real-time reverse transcription-polymerase chain reaction assays.

Genes	Forward primer (5′ → 3′)	Reverse primer (5′ → 3′)	Accession
β -actin	CATTGCTGACAGGATGCAGAAGG	TGCTGGAAGGTGGACAGTGAGG	XM_030254057.1
Gal-1	GTAACACCAAGGAAGATGGGACC	TCATGTCCGTCTGGCAGCTTGA	NM_008495.2
Gal-3	GGAGAGGGAATGATGTTGCCT	TCCTGCTTCGTGTTACACACA	NM_010705.3
Gal-9	CTGGAATCCCTCCTGTGGTGTA	CCTCGTAGCATCTGGCAAGACA	NM_001159301.1
ECP	CATCACCAGTCGGAGGAGAACA	ATGGGACTGTCCTGTGGAGTTC	XM_021155370.1
EPO	CTGTCTCCTGACTAACCGCTCT	TCAGCGGCTAGGCGATTGTGTT	XM_006532174.3
MBP	CAAGACCTGTCGCTACCTCCTA	GCGGACTGGATTCCGAAGTTAAC	XM_021156862.1
MBP	GATGAGGCTTCCTGTCCCTACT	TGACAGGTTTTGGAATAGCATTTCC	NM_010558.1
TGF-β	TGATACGCCTGAGTGGCTGTCT	CACAAGAGCAGTGAGCGCTGAA	XM_021167684.1
CCL11	TCCATCCCAACTTCCTGCTGCT	CTCTTTGCCCAACCTGGTCTTG	NM_011330.3
CCR3	CCACTGTACTCCCTGGTGTTCA	GGACAGTGAAGAGAAAGAGCAGG	XM_017313120.2
CCL24	ATTCCAGAAAACCGAGTGGTTAGC	GCATCCAGTTTTTGTATGTGCCTC	NM_019577.5
α-SMA	TGCTGACAGAGGCACCACTGAA	CAGTTGTACGTCCAGAGGCATAG	XM_021152572.2
Collagen 1	CCTCAGGGTATTGCTGGACAAC	CAGAAGGACCTTGTTTGCCAGG	XM_021213774.2

### Statistics

Results of experimental studies were reported as mean ± SD. Statistical analysis of the data was performed by one-way ANOVA followed by LSD-*t* multiple comparison tests using SPSS software for windows (version 25.0; SPSS, Inc., IL, United States). Pearson’s correlation coefficient was used to analyze correlations between the levels of galectins and chemokines or eosinophil granule proteins. The graphs were performed using SPSS software for windows and GraphPad Prism 7 software (GraphPad Software, La Jolla, CA, United States). A value of *P* < 0.05 was considered significant.

## Results

### Blockage of Galectin-Receptor Interactions Promoted the Heart Pathology and Eosinophil Infiltration of *T. spiralis*-Infected Mice

**Figure 1:**
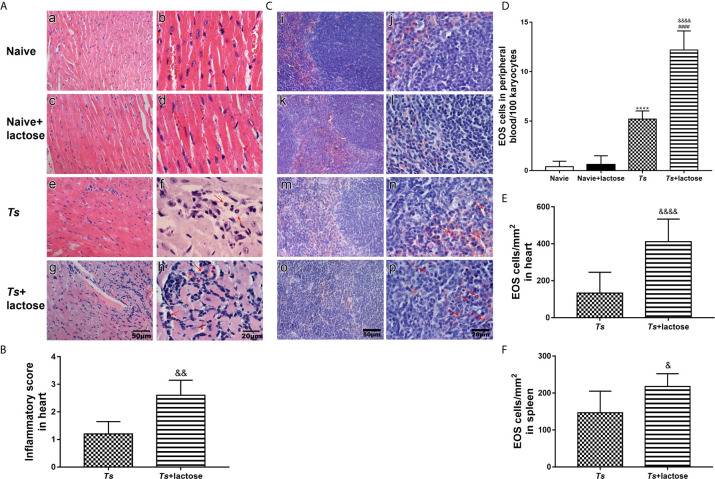
Histopathology and eosinophil counts in different groups of mice. Histopathology of heart **(A)** and spleen **(C)**. No histopathological changes and eosinophils were observed in the heart (a, b) and spleen (i, j) tissues of uninfected mice and in the heart (c, d) and spleen (k, l) tissues of uninfected mice with α-lactose treatment. Histopathological changes and obvious inflammation were observed in the heart (e, f) and spleen (m, n) tissues of *T. spiralis*-infected mice and in the heart (g, h) and spleen (o, p) tissues of *T. spiralis*-infected mice plus α-lactose treatment. Eosinophils indicated by red arrows were observed in the heart and spleen tissues of *T. spiralis*-infected mice and *T. spiralis*-infected mice plus α-lactose treatment. Original magnification 400× (scale bar = 50 µm) for a, c, e, g, i, k, m, and o; 1000× (scale bar = 20 µm) for b, d, f, h, j, l, n, and p; H&E stain. **(B)** Histopathological score analysis of the heart. **(D)** The eosinophil (EOS) count in the peripheral blood. Quantitative analysis of eosinophils in the heart **(E)** and spleen **(F)**. The density of eosinophils was expressed as the number of eosinophils per square millimeter. Data are presented as means ± SD; there were eight mice in each group and the data shown are representative of those from two different experiments. ****P < 0.0001 *vs*. uninfected control mice. ^####^ *P* < 0.0001 *vs*. uninfected mice or uninfected mice with α-lactose treatment. ^&^ *P* < 0.05, ^&&^ *P* < 0.01, and ^&&&&^ *P* < 0.0001 *vs*. *T. spiralis*-infected mice.

Histological observation showed that the sections of heart from uninfected mice and uninfected mice with α-lactose treatment had no inflammation or structural abnormality. Inflammation and eosinophil infiltration were observed in the heart tissues of *T. spiralis-*infected mice; however, more severe inflammation and more eosinophil infiltration were observed in the heart tissues of *T. spiralis*-infected mice plus α-lactose treatment (**Figure 1A**). Semi-quantitative analysis of the severity of inflammation in the heart sections between the two infected groups was performed. Compared with *T. spiralis*-infected mice, the histopathological score was significantly increased in the heart tissues of *T. spiralis*-infected mice with α-lactose treatment (*P* < 0.01) (**Figure 1B**). Histological observation showed that the sections of spleen from uninfected mice and uninfected mice with α-lactose treatment had no inflammation or structural abnormality. However, mild inflammation and eosinophil infiltration were observed in the spleen tissues of *T. spiralis-*infected group, but severe inflammation and increased eosinophil infiltration were observed in the spleen tissues of *T. spiralis*-infected mice plus α-lactose treatment (**Figure 1C**).

The numbers of eosinophils in peripheral blood, and heart and spleen tissues of different groups under 1000× magnification were counted. Eosinophil numbers in the peripheral blood were significantly increased in both *T. spiralis*-infected mice and *T. spiralis*-infected mice plus α-lactose treatment compared with uninfected mice or uninfected mice with α-lactose treatment (*P* < 0.0001). When compared with *T. spiralis*-infected mice, eosinophil number was significantly increased in the peripheral blood of *T. spiralis*-infected mice plus α-lactose treatment (*P* < 0.0001) (**Figure 1D**), significantly increased in the heart tissues of *T. spiralis*-infected mice plus α-lactose treatment (*P* < 0.0001) (**Figure 1E**), and significantly increased in the spleen tissues of *T. spiralis*-infected mice plus α-lactose treatment (*P* < 0.05) (**Figure 1F**). The results indicate that blockage of galectin-receptor interactions increases eosinophil numbers in the peripheral blood, heart, and spleen of *T. spiralis*-infected mice.

### Blockage of Galectin-Receptor Interactions Increased Heart Fibrosis in *T. spiralis*-Infected Mice

**Figure 2:**
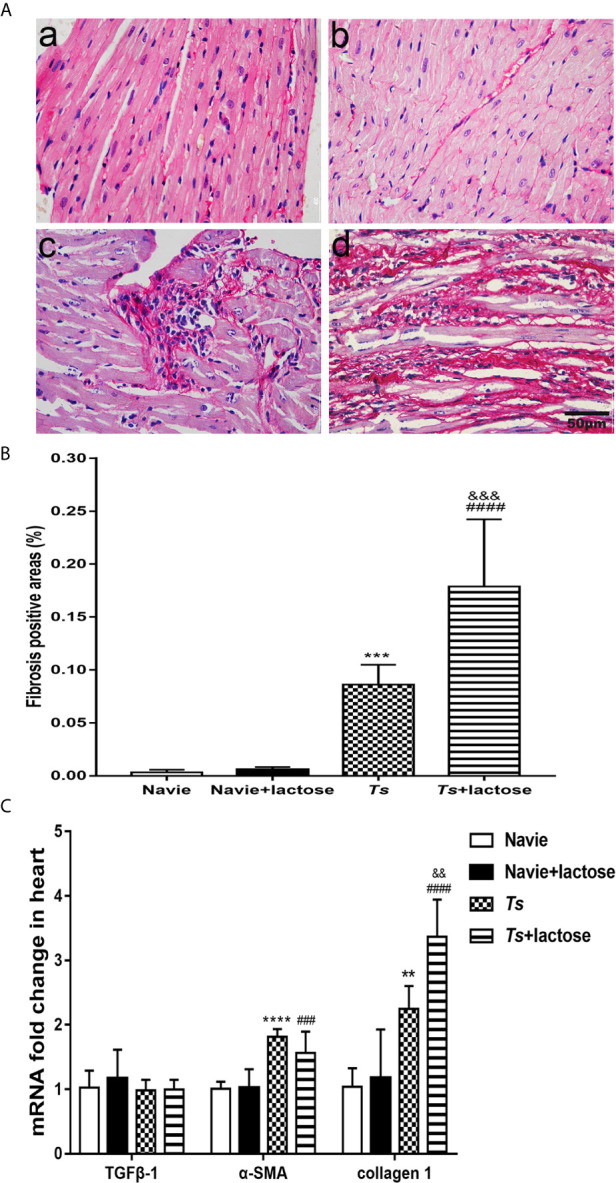
Cardiac fibrosis and mRNA levels of TGFβ-1, α-SMA, and collagen 1 in the hearts of different groups of mice. **(A)** Cardiac fibrosis. No obvious fibrosis was observed in the heart tissues of uninfected mice (a) and uninfected mice with α-lactose treatment (b); severe fibrosis was observed in the heart tissues of *T. spiralis*-infected mice (c) and *T. spiralis*-infected mice with α-lactose treatment (d). Original magnification 400× (scale bar = 50 µm); sirius red stain. **(B)** Quantitative analysis of fibrosis areas in the heart. The positive area of fibrosis was expressed as percentage. **(C)** The mRNA expression levels of TGFβ-1, α-SMA, and collagen 1 in the heart. Data are presented as means ± SD; there were eight mice in each group and the data shown are representative of those from two different experiments. ^**^ *P* < 0.01, ^***^ *P* < 0.001, and ^****^ *P* < 0.0001 vs. uninfected mice. ^###^ *P* < 0.001 and ^####^ *P* < 0.0001 *vs*. uninfected mice or uninfected mice with α-lactose treatment. ^&&^ *P* < 0.01 and ^&&&^ *P* < 0.001 *vs*. *T. spiralis-*infected mice.

Sirius red staining showed that the heart tissues of uninfected mice and uninfected mice with α-lactose treatment had no obvious fibrosis. However, severe fibrosis was observed in the heart tissues of *T. spiralis*-infected mice and more severe fibrosis was observed in the heart tissues of *T. spiralis*-infected mice plus α-lactose treatment (**Figure 2A**). Using Image-Pro Plus 6.0 to determine the percentage of fibrosis positive areas, the results showed that compared with uninfected mice or uninfected mice treated with α-lactose, there were significantly increased fibrosis in the heart tissues of *T. spiralis-*infected mice (*P* < 0.001) and *T. spiralis*-infected mice plus α-lactose treatment (*P* < 0.0001). Compared with *T. spiralis-*infected mice, there was more severe fibrosis in the heart tissues of *T. spiralis*-infected mice plus α-lactose treatment (*P* < 0.001) (**Figure 2B**). The data indicate that galectin-receptor interactions may attenuate cardiac fibrosis caused by *T. spiralis* infection.

The mRNA levels of TGFβ-1, α-SMA, and collagen 1 relative to control group (e.g. the relative transcript level in uninfected group = 1.0) were determined. Compared with uninfected mice or uninfected mice with α-lactose treatment, the expression levels of α-SMA (*P* < 0.0001 and *P* < 0.001, respectively) and collagen 1 (*P* < 0.01 and *P* < 0.0001, respectively) were significantly increased in the hearts of both *T. spiralis*-infected mice and *T. spiralis*-infected mice plus α-lactose treatment. Compared with *T. spiralis*-infected mice, the level of collagen 1 (*P* < 0.01) was significantly increased in the hearts of *T. spiralis*-infected mice plus α-lactose treatment. However, TGFβ-1 level had no significant difference in the hearts among all the groups (**Figure 2C**). The results suggest that α-SMA and collagen 1 may involve in the cardiac fibrosis caused by *T. spiralis* infection.

### Blockage of Galectin-Receptor Interactions Increased Serum Biomarker of Myocardial Damage and Cardiac Function Damage of *T. spiralis*-Infected Mice

**Figure 3:**
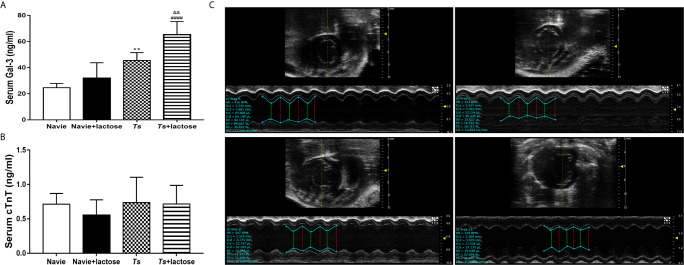
The serum levels of Gal-3 **(A)** and cTnT **(B)**, and the M-mode echocardiography **(C)** of different groups of mice. Uninfected mice (a), uninfected mice with α-lactose treatment (b), *T. spiralis*-infected mice (c), and *T. spiralis*-infected mice with α-lactose treatment (d). ^**^ *P* < 0.01 *vs*. uninfected mice. ^####^ *P* < 0.0001 *vs*. uninfected mice or uninfected mice with α-lactose treatment. ^&&^ *P* < 0.01 *vs*. *T. spiralis-*infected mice.

The cTnT is a sensitive and highly specific marker of myocardial injury (37). In addition, Gal-3 has becoming a powerful predictor of heart failure and mortality (38). Our results showed that serum Gal-3 levels were significantly increased in both *T. spiralis*-infected mice (*P* < 0.01) and *T. spiralis*-infected mice plus α-lactose treatment (*P* < 0.0001) compared with uninfected mice or uninfected mice with α-lactose treatment. Compared with *T. spiralis*-infected mice, serum Gal-3 level was significantly increased in *T. spiralis*-infected mice plus α-lactose treatment (*P* < 0.01) (**Figure 3A**). However, serum cTnT level had no difference among all the groups (**Figure 3B**).

Echocardiography is a non-invasive technique that provides quantitative information on the dimensions, contractile kinetics, as well as tissue and blood velocities in the heart (39). At 30 days of *T. spiralis* infection, *T. spiralis*-infected mice and *T. spiralis*-infected mice plus α-lactose treatment had varying degrees of left ventricular systolic function damage detected by transthoracic conventional echocardiography (**Figure 3C**). Compared with uninfected mice or uninfected mice with α-lactose treatment, there were significantly decreased LVEF (*P* < 0.05 and *P* < 0.0001, respectively) and FS (*P* < 0.05 and *P* < 0.0001, respectively) in the hearts of *T. spiralis*-infected mice and *T. spiralis-*infected mice plus α-lactose treatment, and significantly decreased CO (*P* < 0.01) and SV (*P* < 0.05) in the hearts of *T. spiralis*-infected mice plus α-lactose treatment. However, compared with *T. spiralis*-infected mice, there were significantly decreased LEVF (*P* < 0.001), FS (*P* < 0.001), CO (*P* < 0.05), and SV (*P* < 0.05) in the hearts of *T. spiralis-*infected mice plus α-lactose treatment (**Table 2**). The results suggest that blockage of galectin-receptor interactions may aggravate the cardiac function damage of *T. spiralis*-infected mice.

**Table 2:** The left ventricular systolic function of different groups of mice.

Echocardiographic data	Naive	Naive + lactose	*Ts*	*Ts +* lactose
LVEF%	66.09 ± 2.06	68.66 ± 2.50	61.20 ± 1.01^*^	51.48 ± 1.24^#### &&&^
FS%	35.81 ± 1.64	37.61 ± 1.93	32.22 ± 0.89^*^	25.52 ± 0.52^#### &&&^
SV μL	41.26 ± 7.07	34.00 ± 7.91	39.19 ± 7.93	23.01 ± 3.85^# &^
CO mL/min	19.35 ± 4.98	13.57 ± 2.22	17.37 ± 5.63	7.92 ± 0.75^## &^

^*^P < 0.05 vs. uninfected mice. ^#^P < 0.05, ^##^P < 0.01, and ^####^P < 0.0001 vs. uninfected mice or uninfected mice with α-lactose treatment. ^&^P < 0.05 and ^&&&^P < 0.001 vs. T. spiralis-infected mice.

### Blockage of Galectin-Receptor Interactions Increased the Expression of Gal-3 in the Heart of *T. spiralis*-Infected Mice

**Figure 4:**
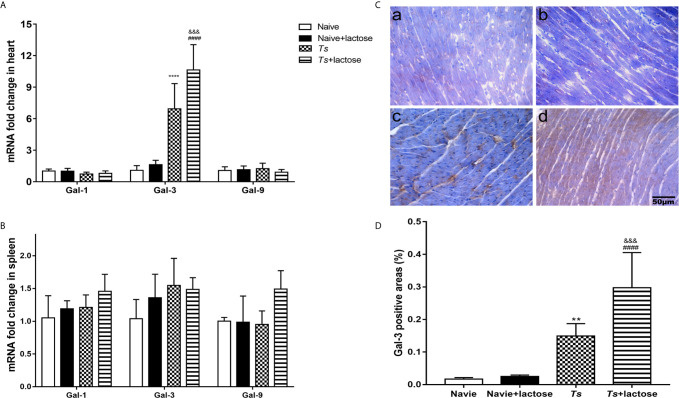
The mRNA levels of Gal-1, Gal-3, and Gal-9 in the hearts **(A)** and spleens **(B)**, and immunohistochemical detection of Gai-3 **(C)** and quantitative analysis of Gal-3 positive areas in the hearts **(D)** of different groups of mice. The mRNA expression values are means from triplicate measurements and data are presented as means ± SD. Immunohistochemistry for Gal-3 (brown color) in the heart tissues of uninfected mice (a), uninfected mice with α-lactose treatment (b), *T. spiralis*-infected mice (c), and *T. spiralis*-infected mice with α-lactose treatment (d). Original magnification 400× (scale bar = 50 µm). The positive area of Gal-3 was expressed as percentage. Data are presented as means ± SD; there were eight mice in each group and the data shown are representative of those from two different experiments. ^**^ *P* < 0.01 and ^****^ *P* < 0.0001 *vs*. uninfected control mice. ^####^ *P* < 0.0001 *vs*. uninfected mice or uninfected mice with α-lactose treatment. ^&&&^ *P* < 0.001 *vs*. *T. spiralis*-infected mice.

The mRNA levels of Gal-1, Gal-3, and Gal-9 in the heart and spleen were determined. Compared with uninfected mice or uninfected mice with α-lactose treatment, Gal-3 expression levels were significantly increased in the hearts of both *T. spiralis*-infected mice and *T. spiralis*-infected mice plus α-lactose treatment (*P* < 0.0001). Compared with *T. spiralis*-infected mice, Gal-3 level was significantly increased in the heart of *T. spiralis*-infected mice plus α-lactose treatment (*P* < 0.001). The levels of Gal-1 and Gal-9 in the heart had no significant difference among all the groups (**Figure 4A**). In addition, the levels of Gal-1, Gal-3, and Gal-9 in the spleen had also no significant difference among all the groups (**Figure 4B**).

Immunohistochemical staining showed that there were few Gal-3 positive cells (brown color) in the heart tissues of uninfected mice and uninfected mice with α-lactose treatment. However, there were obvious Gal-3 positive areas in the hearts of *T. spiralis*-infected mice and *T. spiralis*-infected mice plus α-lactose treatment (**Figure 4C**). Semi-quantitative analysis of Gal-3 positive areas in the heart sections of different groups was performed. Compared with uninfected mice or uninfected mice with α-lactose treatment, the percentages of Gal-3 positive areas were significantly increased in the heart tissues of *T. spiralis*-infected mice (*P* < 0.01) and *T. spiralis*-infected mice plus α-lactose treatment (*P* < 0.0001). When compared with *T. spiralis*-infected mice, the percentage of Gal-3 positive areas was significantly increased in the heart tissues of *T. spiralis*-infected mice plus α-lactose treatment (*P* < 0.001) (**Figure 4D**). The results suggest that Gal-3 is strongly involved in cardiac immunopathology caused by *T. spiralis* infection.

### Blockage of Galectin-Receptor Interactions Increased Extracellular Traps in the Heart of *T. spiralis*-infected Mice

**Figure 5:**
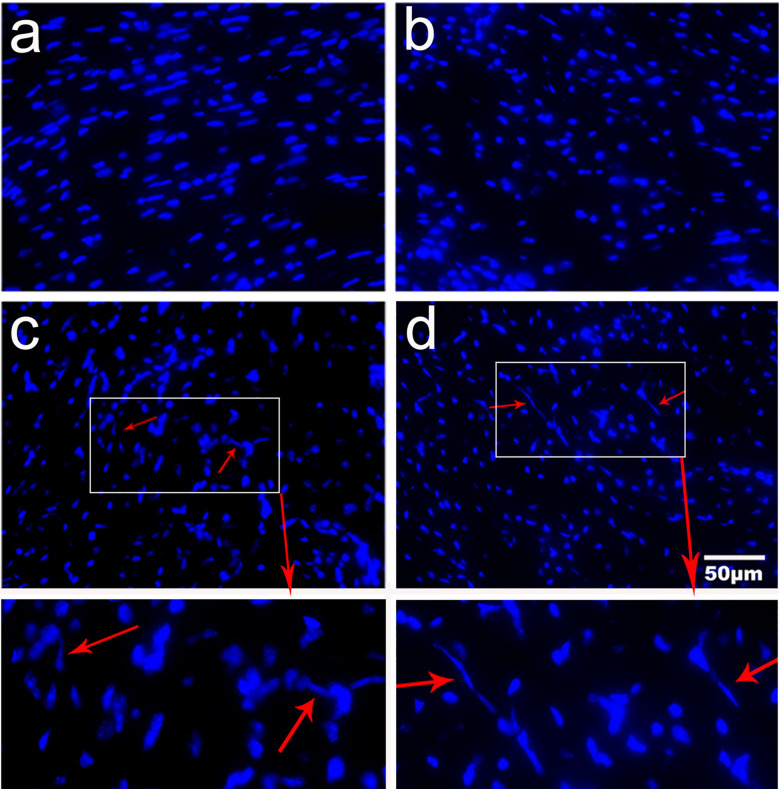
Expression of extracellular traps in the heart tissues of different groups of mice. Fluorescence staining showed no extracellular trap in uninfected mice (a) and uninfected mice with α-lactose treatment (b). Inflammatory cells released DNA extracellular traps (red arrows) were observed in the heart tissues of *T. spiralis*-infected mice (c) and *T. spiralis*-infected mice with α-lactose treatment (d). Original magnification 400× (scale bar = 50 µm).

Immunofluorescence staining revealed that there was no extracellular trap observed in uninfected mice and uninfected mice treated with α-lactose. However, extracellular traps were observed in the heart tissues of both *T. spiralis*-infected mice and *T. spiralis*-infected mice plus α-lactose treatment (**Figure 5**), suggesting that *T. spiralis* infection may induce inflammatory cells to release extracellular traps.

### Blockage of Galectin-Receptor Interactions Increased mRNA Expression of Eosinophil Granule Proteins in the Heart and Spleen of *T. spiralis*-Infected Mice

**Figure 6:**
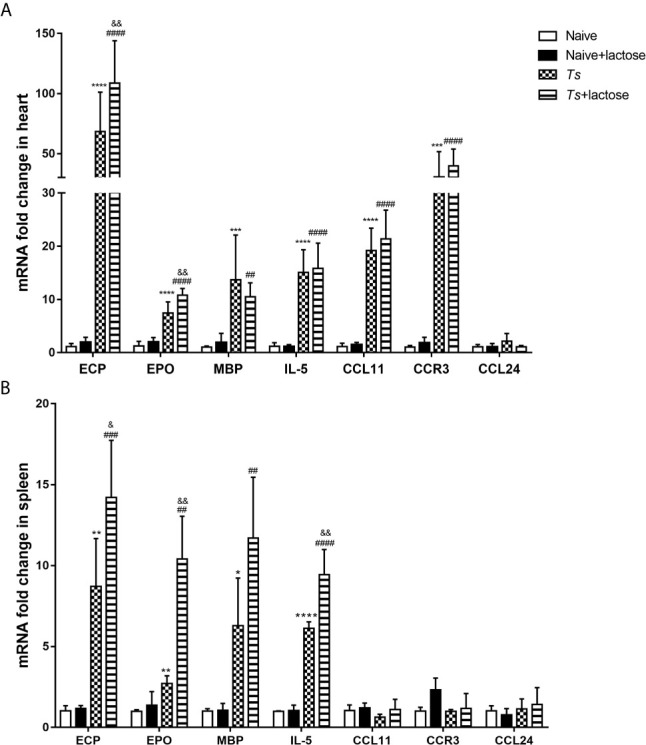
The mRNA levels of ECP, EPO, MBP, IL-5, CCL11, CCR3, and CCL24 in the hearts **(A)** and spleens **(B)** of different groups of mice. Values are means from triplicate measurements and data are presented as means ± SD; there were eight mice in each group and the data shown are representative of those from two different experiments. ^*^ *P* < 0.05, ^**^ *P* < 0.01, ^***^ *P* < 0.001, and ^****^ *P* < 0.0001 vs. uninfected mice. ^##^ *P* < 0.01, ^###^ *P* < 0.001, and ^####^ *P* < 0.0001 *vs*. uninfected mice or uninfected mice with α-lactose treatment. ^&^ *P* < 0.05 and ^&&^ *P* < 0.01 *vs*. *T. spiralis-*infected mice.

The mRNA expression levels of eosinophil chemokines (IL-5, CCL11, CCL24, and CCR3) and eosinophil cationic granule proteins (ECP, EPO, and MBP) in the heart and spleen were determined. Compared with uninfected mice or uninfected mice with α-lactose treatment, the expression levels of ECP (*P* < 0.0001), EPO (*P* < 0.0001), MBP (*P* < 0.001 and *P* < 0.01, respectively), IL-5 (*P* < 0.0001), CCL11 (*P* < 0.0001), and CCR3 (*P* < 0.001 and *P* < 0.0001, respectively) were significantly increased in the hearts of *T. spiralis*-infected mice and *T. spiralis*-infected mice plus α-lactose treatment. Compared with *T. spiralis*-infected mice, the levels of ECP and EPO were significantly increased in the heart of *T. spiralis*-infected mice plus α-lactose treatment (*P* < 0.01) (**Figure 6A**).

Compared with uninfected mice or uninfected mice with α-lactose treatment, the expression levels of ECP (*P* < 0.01 and *P* < 0.001, respectively), EPO (*P* < 0.01), MBP (*P* < 0.05 and *P* < 0.01, respectively), and IL-5 (*P* < 0.0001) were significantly increased in the spleen of *T. spiralis*-infected mice and *T. spiralis*-infected mice plus α-lactose treatment. Compared with *T. spiralis*-infected mice, the levels of ECP (*P* < 0.05), EPO (*P* < 0.01), and IL-5 (*P* < 0.01) were significantly increased in the spleen of *T. spiralis*-infected mice plus α-lactose treatment (**Figure 6B**). The results indicate that blockage of galectin-receptor interactions may increase eosinophil activation in both hearts and spleens of *T. spiralis-*infected mice.

### Correlations Between Gal-3 and Eosinophil Granule Proteins, α-SMA, or Collagen 1

**Figure 7:**
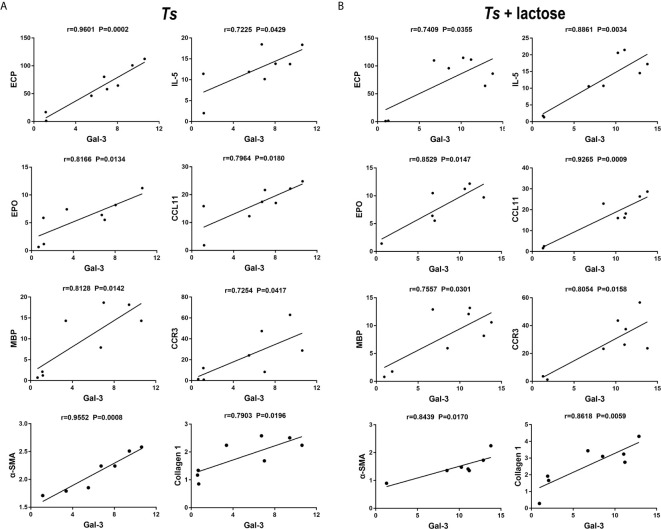
Correlation analysis between the mRNA expression levels of Gal-3 and ECP, EPO, MBP, IL-5, CCL11, CCR3, α-SMA, or collagen 1 in the heart of *T. spiralis-*infected mice **(A)** and *T. spiralis*-infected mice with α-lactose treatment **(B)**. The *r* value generates the theoretical line of best fit, and the *P* value indicates the significance of the correlation. There were eight mice in each group and the data shown are representative of those from two different experiments.

The correlations between the mRNA levels of Gal-3 and eosinophil granule proteins, α-SMA, or collagen 1 in the heart of *T. spiralis*-infected mice and *T. spiralis*-infected mice with α-lactose treatment were evaluated. Only significant correlations were provided here. In *T. spiralis*-infected mice, there were significant correlations between the mRNA levels of Gal-3 and ECP (*r* = 0.9601, *P* = 0.0002), Gal-3 and EPO (*r* = 0.8166, *P* = 0.0134), Gal-3 and MBP (*r* = 0.8128, *P* = 0.0142), Gal-3 and IL-5 (*r* = 0.7225, *P* = 0.0429), Gal-3 and CCL11 (*r* = 0.7964, *P* = 0.0180), Gal-3 and CCR3 (*r* = 0.7254, *P* = 0.0417), Gal-3 and α-SMA (*r* = 0.9552, *P* = 0.0008), and Gal-3 and collagen 1 (*r* = 0.7903, *P* = 0.0196) in the heart (**Figure 7A**). In *T. spiralis*-infected mice plus α-lactose treatment, there were significant correlations between the mRNA levels of Gal-3 and ECP (*r* = 0.7409, *P* = 0.0355), Gal-3 and EPO (*r* = 0.8529, *P* = 0.0147), Gal-3 and MBP (*r* = 0.7557, *P* = 0.0301), Gal-3 and IL-5 (*r* = 0.8861, *P* = 0.0034), Gal-3 and CCL11 (*r* = 0.9265, *P* = 0.0009), Gal-3 and CCR3 (*r* = 0.8054, *P* = 0.0158), Gal-3 and α-SMA (*r* = 0.8439, *P* = 0.0170), and Gal-3 and collagen 1 (*r* = 0.8618, *P* = 0.0059) in the heart (**Figure 7B**).

## Discussion

It has been reported that acute myocardial infarction caused by *T. spiralis* infection revealed multiple necroses and fibroses of the myocardium in a patient (40). Rats infected with *T. spiralis* showed that during acute myocarditis (from day 21 p.i. onwards), the immunopathological reactions may have a role in the induction of myocardial damage and dysfunction; eosinophils and mast cells appear to undergo degranulation (41). In this paper, our data showed that *T. spiralis* infection can induce cardiac pathology and dysfunction in Kunming mice, suggesting that Kunming mice may be a suitable animal model for the study of *T. spiralis*-associated myocarditis.

Galectins are important modulators participating in heart inflammation and cardiovascular disease, and Gal-3 is the major galectin expressed in the heart (21). Using murine models of *Trypanosoma cruzi* infection, Pineda et al. (42) reported that lack of Gal-3 prevents cardiac fibrosis and effective immune responses in *T. cruzi* experimental infection; however, conflicting results demonstrated the beneficial impact of Gal-3 expression to the control of infection and to limit heart tissue damage during *T. cruzi* infection (43). In the present study, we demonstrated that Gal-3 level was significantly increased in the hearts measured by either qRT-PCR assay or immunohistochemical staining, accompanied with increased inflammation and fibrosis in the myocardium of *T. spiralis*-infected mice, and higher Gal-3 levels were examined in the heart of *T. spiralis*-infected mice with α-lactose treatment. In addition, fibrosis markers (α-SMA and collagen I) may play an important role in the cardiac fibrosis caused by *T. spiralis* infection.

It has been reported that cTnT is a marker of myocyte injury (37) and Gal-3 is a prognostic marker of heart failure (38). In the present study, the serum cTnT level had no change after *T. spiralis* infection. Although the serum Gal-3 levels were significantly increased in both *T. spiralis*-infected groups, there was even higher serum Gal-3 level in *T. spiralis*-infected mice plus α-lactose treatment. Echocardiography showed that the left ventricular systolic function was decreased in both *T. spiralis*-infected groups; however, more severe cardiac function damage in *T. spiralis*-infected mice plus α-lactose treatment. Our data demonstrated that blockage of galectin-receptor interactions may aggravate heart function damage caused by *T. spiralis* infection, which are coincided with the severe myocardial inflammation and fibrosis in this group.

*T. spiralis* induces a pronounced eosinophilia that coincides with establishment of larval stages in skeletal muscle (44). In the present study, it showed that eosinophils increased in the peripheral blood, heart, and spleen of *T. spiralis*-infected mice and *T. spiralis*-infected mice plus α-lactose treatment. The mRNA levels of IL-5, CCL11, and CCR3 were increased in the heart of either *T. spiralis*-infected mice or *T. spiralis*-infected mice plus α-lactose treatment, indicates that IL-5, CCL11, and CCR3 may promote migration and activation of eosinophils. Eosinophils play a vital role in defense against parasites and they are recruited to the inflamed area in response to stimuli and chemotactic factor, IL-5, an essential cytokine in eosinophil development, can promote the eosinophils terminal differentiation and activation (45). CCL11 is a potent chemokine that promotes migration and activation of eosinophils (46). CCL11 and its receptor, CCR3, play an important pathophysiological role in the accumulation of eosinophils and neutrophils as well as in the production of fibrogenic cytokines during bleomycin-induced lung injury and fibrosis (47). When eosinophils are recruited to the inflammation area, they are activated and release cytokines and cationic proteins, which modulate the immune response. ECP is present in eosinophil granules and has been associated with eosinophil-associated disorders (48). EPO is exclusively synthesized and released by eosinophils (49). In the present study, the infiltration of eosinophils was more intense in the sections of myocardium and spleen of *T. spiralis*-infected mice with α-lactose treatment than that of *T. spiralis*-infected mice. In addition, there were significantly increased ECP, EPO, and IL-5 mRNA expressions in the heart and spleen of *T. spiralis*-infected mice with α-lactose treatment. Positive correlations were found in the mRNA levels between Gal-3 and ECP, EPO, MBP, IL-5, CCL11, CCR3, α-SMA, or collagen 1 in the hearts of both *T. spiralis*-infected mice and *T. spiralis*-infected mice with α-lactose treatment, suggesting that Gal-3 may promote eosinophil infiltration into the heart and results in subsequent cardiac fibrosis and functional damage in *T. spiralis*-infected mice.

Extracellular traps are produced by several immune cells including neutrophils (50), eosinophils (51), mast cells (52), and monocytes/macrophages in humans and mice (53). In this study, our *in vivo* results demonstrate that mice do produce extracellular traps in response to infection with *T. spiralis*. However, whether the extracellular traps are associated with trapping and killing the *T. spiralis* larvae *in vivo* remain to be further studied.

In conclusion, in the present study, eosinophils increase in the peripheral blood, and accumulate in the heart and spleen through induction of IL-5, CCL11, and CCR3, and release ECP, EPO, and MBP by degranulation, which may cause eosinophilic myocarditis in *T. spiralis*-infected mice. However, blockage of galectin-receptor interactions further promotes Gal-3 production and eosinophil infiltration in the heart of *T. spiralis*-infected mice, accompanied with exacerbated cardiac immunopathology, collagen deposition, fibrosis, and subsequent left ventricular systolic function damage. Our data indicate that Gal-3 may have a harmful effect on the heart during acute *T. spiralis* infection in a murine model.

## Data Availability Statement

The original contributions presented in the study are included in the article/supplementary material. Further inquiries can be directed to the corresponding authors.

## Ethics Statement

Animal studies were conducted according to protocols approved by the Animal Experimentation Ethics Committee of Zhongshan School of Medicine on Laboratory Animal Care at Sun Yat-sen University, China.

## Author Contributions

FL conceived and designed the experiments, analyzed the data, and wrote the manuscript. JY performed experiments and analyzed data. SH edited the manuscript. All authors contributed to the article and approved the submitted version.

## Funding

This work was supported by the Natural Science Foundation of China (nos. 81971955 and 81471973), the Natural Science Foundation of Guangdong Province, China (nos. 2019A1515011667 and 2021A1515012115), the open project of Key Laboratory of Tropical Disease Control of Ministry of Education, Sun Yat-sen University, China (no. 2020ZX02), and the undergraduate teaching quality engineering project of Sun Yat-sen University, China (SYSU Undergraduate Education [2021] 93).

## Conflict of Interest

The authors declare that the research was conducted in the absence of any commercial or financial relationships that could be construed as a potential conflict of interest.
